# Electromechanical Response of High-Performance Fiber-Reinforced Cementitious Composites Containing Milled Glass Fibers under Tension

**DOI:** 10.3390/ma11071115

**Published:** 2018-06-29

**Authors:** Min Kyoung Kim, Dong Joo Kim

**Affiliations:** Department of Civil and Environmental Engineering, Sejong University, 209, Neungdong-ro, Gwangjin-gu, Seoul 05006, Korea; mkkim9112@naver.com

**Keywords:** high-performance fiber-reinforced cementitious composites (HPFRCCs), self-damage sensing, milled glass fibers (MGFs), electrical resistivity, interfacial bond strength

## Abstract

The self-damage sensing capacity of high-performance fiber-reinforced cementitious composites (HPFRCCs) that blended long- (1 vol %) and medium-length (1 vol %) smooth steel fibers was considerably improved by adding milled glass fibers (MGFs) with a low electrical conductivity to a mortar matrix. The addition of MGFs (5 wt %) significantly increased the electrical resistivity of the mortar matrix from 45.9 to 110.3 kΩ·cm (140%) and consequently improved the self-damage sensing capacity (i.e., the reduction in the electrical resistivity during the tensile strain-hardening response) from 17.27 to 25.56 kΩ·cm (48%). Furthermore, the addition of MGFs improved the equivalent bond strength of the steel fibers on the basis of the higher pullout energy owing to the accumulated cementitious material particles attached to the surfaces of steel fibers.

## 1. Introduction

Structural health monitoring (SHM) has played a very important role in protecting human lives and the assets of human society from the catastrophic structural collapses associated with the early deterioration of construction materials. At present, SHM mostly utilizes attached and/or embedded sensors; however, their durability is extremely low, especially compared with the long-term service lives of buildings or civil infrastructure, and their sensing area is very limited [1,2].

To overcome these limitations of the sensors used in present SHM systems, much research on the development of smart construction materials with self-sensing capacity has been conducted during the last two decades [3,4,5], although the electromechanical response of cement-based composites under flexure was first reported by Wittmann [6] in 1973.

In order to develop the self-sensing capacity by utilizing the electromechanical response of cement-based composites under various loading conditions, much research has focused on enhancing the electrical conductivity of such composites by adding functional fillers, e.g., carbon fibers, carbon nanofibers, carbon nanotubes, carbon black, and graphite [3,7,8,9,10]. Cement-based composites containing carbon materials have demonstrated an excellent self-strain or -damage sensing capacity and produced superior mechanical properties including a high strength and toughness [11,12,13,14,15,16,17,18,19,20,21,22,23]. The gauge factor (approximately 60) of continuous carbon fiber-reinforced cement composites (CFRCs) is 30 times greater than that (approximately 2) of conventional strain gages [24]. Furthermore, cement-based composites containing short carbon fibers have produced much higher gauge factors (up to 700) under compressive, tensile, and flexural loads [25]. Cement-based composites containing nanomaterials, e.g., carbon nanotubes and carbon nanofibers, have also demonstrated a high self-strain or -damage sensing capacity [4,14,15,19,20,21,22,26,27,28,29,30,31,32], even though achieving a uniform distribution of nanomaterials within cement-based composites remains quite challenging [33]. The distribution of nanomaterials could be enhanced by using surfactants, sonicators, and/or mechanical mixing procedures [10,12,15,22,27,30,34,35,36]. However, the self-sensing capacity of cement-based composites containing carbon materials is still limited within their elastic region owing to their brittle failure [34,37,38].

Steel fiber-reinforced cement composites (SFRCs) have also demonstrated self-sensing capacity under various loading conditions owing to the high electrical conductivity of the steel fibers [39,40,41,42,43,44]. The gauge factors of SFRCs under compression and tension were reported to be approximately 200 and 4560, respectively, while those of CFRCs were 350 and 90, respectively [39,45]. Recently, several studies [41,42,43,44] have reported the self-damage sensing capacity under tension of high-performance fiber-reinforced cementitious composites (HPFRCCs) containing steel fibers. The volume content and geometry of steel fibers significantly influenced the self-damage sensing capacity [41,42]. Kim et al. [44] also demonstrated that the self-sensing capacity could be enhanced by applying ultra-high-performance concrete (UHPC) with a high electrical resistivity owing to the very low water:cement ratio and densified microstructure. However, UHPCs are very expensive in comparison to normal concrete or SFRCs [46]. Thus, it is necessary to develop the self-sensing HPFRCCs at a lower cost by utilizing relatively economical materials.

This study aims to further enhance the self-damage sensing capacity of HPFRCCs, using relatively low-cost methods, by adding milled glass fibers (MGFs) to the mortar matrix. The addition of MGFs, which have a low electrical conductivity, is expected to increase the electrical resistivity of the mortar matrix, consequently resulting in a more pronounced reduction in the electrical resistivity when inducing crack formation in matrices. The specific objectives are: (1) to achieve a uniform distribution of MGFs in a mortar matrix, (2) to find the optimal amounts of MGFs for maximizing the self-damage sensing capacity of HPFRCCs, and (3) to characterize the electromechanical response of HPFRCCs containing MGFs.

## 2. Electromechanical Response of HPFRCCs under Tension

**Figure 1:**
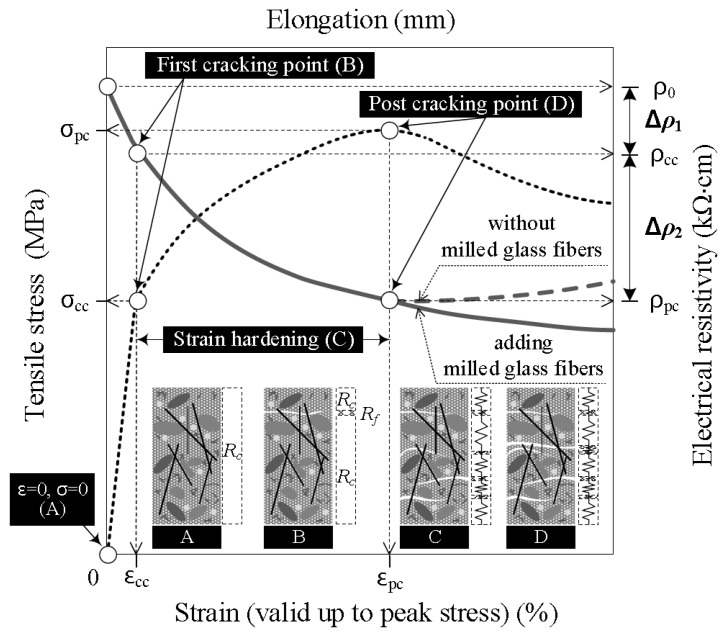
Typical electromechanical response of high-performance fiber-reinforced cementitious composites (HPFRCCs) under tension.

HPFRCCs are typically characterized by unique strain-hardening behavior under direct tension, accompanied by the formation of multiple micro-cracks, as shown in Figure 1. HPFRCCs have demonstrated a much higher strength, ductility, and energy absorption capacity compared with normal concrete or SFRCs. Moreover, the self-damage sensing capacity of HPFRCCs (see Figure 1) can be described as follows: As the tensile strain (*ε*) increases from 0 at Point A to *ε_cc_* at Point B, the tensile stress (*σ*) of HPFRCCs linearly increases from 0 to *σ_cc_*, whereas the electrical resistivity (*ρ*) decreases from the initial electrical resistivity (*ρ*_0_) at Point A to that at the occurrence of the first crack (*ρ_cc_*) at Point B. The reduction in the electrical resistivity until Point B is represented as Δ*ρ*_1_ (Figure 1). During the strain-hardening following the occurrence of the first crack in Range C, as the tensile strain increases from *ε_cc_* at Point B to *ε_pc_* at Point D, the tensile stress further increases from *σ_cc_* to *σ_pc_*, whereas the composite electrical resistivity of the HPFRCCs shows a notable decrease from *ρ_cc_ to ρ_pc_*. The reduction in the electrical resistivity during strain-hardening in Range C is represented by Δ*ρ*_2_ (Figure 1). The total reduction in the electrical resistivity is represented by Δ*ρ*.

The electrical resistance of a composite (*R*) comprises that of both the non-cracked (*R_c_*) and cracked (*R_f_*) parts of the composite [41,42,44], as described in Equation (1) and illustrated in Figure 1. As the number of micro-cracks (*n_cr_*) increases during strain-hardening (Range C in Figure 1), *R_c_* would decrease along with the decreasing total length of the non-cracked part (shown in Figure 1), whereas *R_f_* would increase along with the increasing length of the cracked parts of the composites under tension (Δ*L_debond_*). As *R_f_* is much lower than *R_c_*, the electrical resistance (*R*) of the specimen within the gauge length consequently decreases as the number of multiple micro-cracks increases.

$$R_{c}=\rho_{c}\frac{L-n_{cr}\Delta L_{debond}}{A_{c}}$$

(1) $$R_{f}=\rho_{f}\frac{n_{cr}\Delta L_{debond}}{A_{f}V_{f}}$$

Here, *A_c_* is the cross-sectional area of the composite, *ρ_c_* is the electrical resistivity of the composite, *A_f_* is the area of the steel fibers, *ρ_f_* is the electrical resistivity of a steel fiber, and *V_f_* is the volume content of steel fibers.

The reduction in the electrical resistance (Δ*R*) due to matrix cracking until the post-cracking point can be calculated using Equation (2), since the electrical resistivity of the steel fibers in *R_f_* is much lower than that in *R_c_*, as given by Equation (1) [43,44]:

(2) $$\Delta R=\frac{n_{cr}\Delta L_{debond}}{A_{c}}(\rho_{c}-\frac{\rho_{f}}{V_{f}}).$$

**Figure 2:**
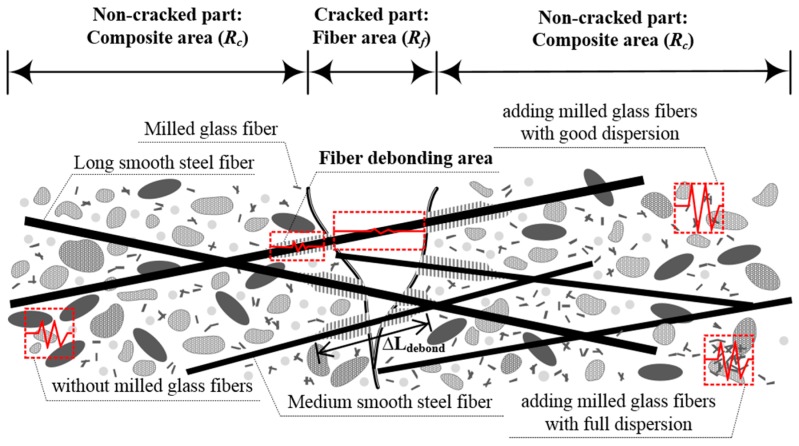
Schematic of HPFRCCs under tension.

As can be seen in Equation (2) and Figure 2, to further increase the self-damage sensing capacity of HPFRCCs under tension, the value of *ρ_c_* should by further increased, in this case by adding MGFs. Thus, in this study, we added MGFs, which have very low electrical conductivity, to the mortar matrix in order to further enhance the electrical resistivity of the composite (*ρ_c_*).

## 3. Experimental Program

**Figure 3:**
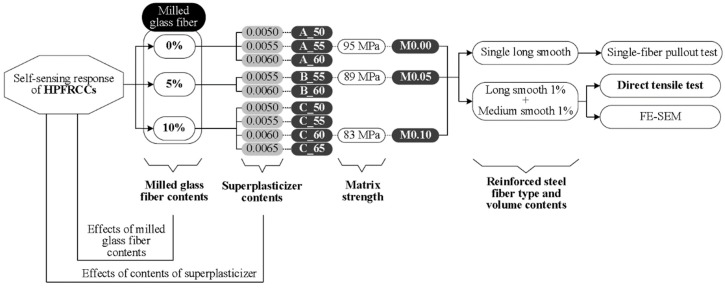
Experimental program.

Figure 3 shows the experimental program designed to investigate the electromechanical response of MGF-containing HPFRCCs under tension. As shown in Table 1, the amount of MGFs varied from 0 to 10 wt % of cement in the matrix composition. The compressive strength (*f’_ck_*) and electrical resistivity (*ρ_m_*) of the mortar matrices, in Table 1, with an optimal amount of superplasticizer, were averaged at least from three specimens. Corresponding to the amount of MGFs added, the amount of superplasticizer was adjusted to facilitate a uniform distribution of MGFs in the matrix by preventing segregation while maintaining a suitable workability. The slump and slump flow of the mortar mixture were also measured. Then, the electromechanical response of the MGF-containing HPFRCCs under tension was investigated by measuring the direct current (DC) electrical resistance of the specimens during direct tensile tests. Single-fiber pullout tests and field-emission scanning electron microscopy (FE-SEM, Model S-4700; Hitachi, Tokyo, Chiyoda-ku, Japan) were utilized to investigate how the addition of MGFs affected the interfacial bond characteristics of steel fibers embedded in mortar matrices.

### 3.1. Materials and Specimen Preparation

**Figure 4:**
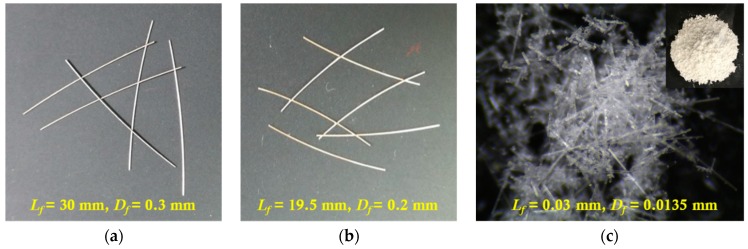
Geometries of the fibers: (**a**) long smooth-, (**b**) medium smooth-steel fibers, and (**c**) MGFs.

Table 2 provides the physical properties of the fibers used in the experiments, while Table 3 lists the chemical components of MGFs. The length and diameter of the long smooth fibers are 30 and 0.3 mm, respectively, while those of the medium-length smooth steel fibers are 19.5 and 0.2 mm, respectively (Figure 4a,b). The MGFs have an average length and diameter of 0.3 and 0.0135 mm, respectively, as shown in Figure 4c. The average grain diameter of silica sand is 0.43 mm, while the superplasticizer contains 25% solid content. 

**Figure 5:**
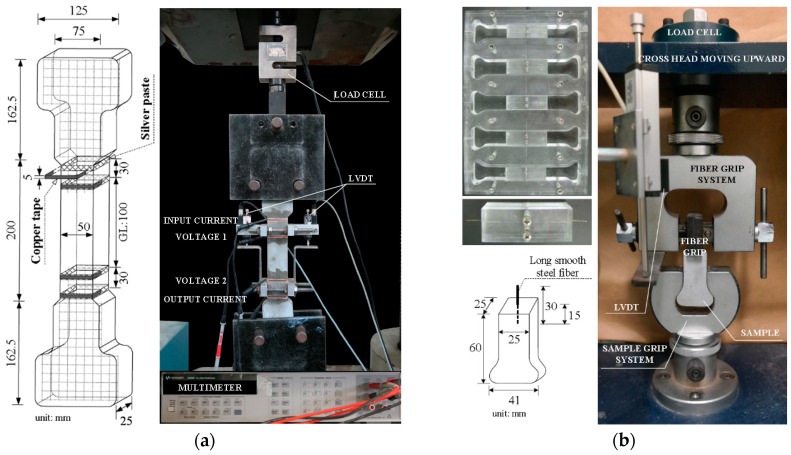
Geometries of the specimens and the test setups: (**a**) direct tensile test and (**b**) single-fiber pullout test.

A Hobart-type laboratory mixer (capacity: 20 L) was used to mix the mortar. Cement, silica sand, and fly ash were first dry-mixed for 3 min and 30 s. Then, MGFs were added to the experimental mortar mixtures and dry-mixed for a further 3 min to ensure their uniform distribution. Water was added to the mixture and mixed for a further 4 min. A superplasticizer was added to the mixture and then stirred for 7 min. For direct tensile specimens, medium smooth steel fibers (1 vol %) were added first; then, long smooth steel fibers (1 vol %) were carefully distributed within the mortar mixture by hand. Two layers of steel wire mesh were reinforced at both ends of the tensile specimens to prevent failure outside the gauge length, as shown in Figure 5a. When the mortar mixture containing MGFs and steel fibers showed a suitable workability, it was poured into molds to produce tensile specimens. At least three specimens were prepared for each series.

For single-fiber pullout specimens, a fiber was first installed in a fiber-holding device to maintain a consistent embedment length (15 mm) and inclination angle (90°) of the fiber, as seen in Figure 5b. The mortar mixtures containing MGFs were then poured into molds to produce samples for single-fiber pullout tests. After casting, all specimens were covered with plastic sheets and placed in a laboratory at room temperature (25 °C) and 60% relative humidity for one day prior to demolding. After demolding, the specimens were water-cured at 24 °C for 14 days. Finally, following the direct tensile tests, samples with a diameter of 24 mm were extracted from both ends of the tensile specimens for FE-SEM analysis and underwent epoxy cold-mounting with a specimen diameter of 30 mm, followed by grinding and polishing (as previously described in Reference [47]). After grinding and polishing, the surfaces of the specimens for FE-SEM analysis were coated with platinum.

To measure the electrical resistivity of the tensile specimens, after curing, a layer of silver paste was applied to the surface of the specimen; then, copper tape, as the electrodes, was attached to the silver paste (see Figure 5a). The distance between the two outer electrodes for the input current (50 μA) was 160 mm, whereas that between the two inner electrodes for voltage measurement was 100 mm, as shown in Figure 5a. Each series comprised at least three specimens, which were stored in a chamber at a constant temperature (25 °C) and relative humidity (60%) prior to testing.

### 3.2. Test Setup and Procedure

The slump flow and slump of the mortar and HPFRCCs containing MGFs were measured in accordance with the respective Korean Industrial Standards, KS F 2594 and KS F 2402. After placing the slump cone horizontally on a flat plate, the cone was filled with the mortar mixture or HPFRCCs containing MGFs. After 30 s, the slump flow was measured as the diameter of the mixture, while the slump was measured as the reduced height of the mixture.

A universal test machine (UTM) with a 300-tonf capacity was used for direct tensile tests, while a UTM with a 500-kgf capacity was used for single-fiber pullout tests. During the tests, the loading speed and data frequency was maintained at 1 mm/min and 5 Hz, respectively. During the tensile tests, the applied load was obtained from a load cell (capacity: 5 tons) located on the top of the specimen, as shown in Figure 5a. The tensile elongation of the specimens was measured by two linear variable differential transformers (LVDTs), while their electrical resistivity was measured using an electrical multimeter (Model 3458A; Keysight, Santa Rosa, CA, USA). Prior to tensioning the specimens, the electrical resistivity was stabilized for at least 20 min to minimize the effects of electrical polarization. During the tensile tests, the temperature and humidity in the laboratory were 9.8 ± 6.9 °C and 30.5% ± 1%, respectively. After direct tensile tests, the equivalent number of multiple micro-cracks of all specimens was calculated by measuring the length of all micro-cracks, which was determined in this study by using Vernier calipers and dividing the total length of micro-cracks by the width of the specimen (50 mm). The length of the micro-crack would be more efficiently calculated by using image analysis techniques later [48,49]. During the single-fiber pullout tests, the pullout load was obtained from the load cell attached to the top of the cross-head, while the slip was measured from the LVDT, as shown in Figure 5b. 

FE-SEM (model SU8010; Hitachi, Tokyo, Japan) was used to investigate the microstructures of HPFRCCs containing MGFs (accelerating voltage: 15 kV; image resolution: 1.0 nm). After coating with platinum, the three samples for FE-SEM analysis were stored in a vacuum chamber prior to capturing the images. The interfacial transition zone (ITZ) and pore images were obtained by FE-SEM; and width of the ITZ (between the steel fiber and mortar matrix) and pore sizes were measured in FE-SEM software from at least four different locations per sample.

## 4. Results

The suitable amounts of superplasticizer for the uniform distribution of MGFs and steel fibers were first determined based on the slump and slump flow tests. The electrical resistance of HPFRCCs is greatly influenced by the distribution of electrically conductive fillers or fibers [15,27,29,31]; thus, the suitable slump flow and slump of HPFRCCs should be carefully determined for the uniform distribution of fillers and fibers because the distribution of fibers is dependent upon the workability of SFRCs [50]. Moreover, the HPFRCC slump was greatly influenced by the accumulation of fibers, whereas the slump flow was more affected by the viscosity and flowability of the matrix and by the distribution of fibers.

Also, the electromechanical response of HPFRCCs was investigated to evaluate the self-sensing capacity of HPFRCCs under tension. The electrical resistivity (*ρ*) can be calculated from the measured electrical resistance (*R*) by using Equation (3). *ρ* is a material property, whereas *R* is affected by the cross-sectional area and the distance between the electrodes.

(3) $$\rho =R\cdot \frac{A}{L}\;\left[\mathrm{units}:\;k\Omega \cdot \mathrm{cm}\right]$$

Here, *A* is the cross-sectional area of the specimen (cm^2^) and *L* is the distance between the two inner electrodes (cm).

As can be seen in the following equations, all of the mechanical parameters, including *σ_pc_*, *ε_pc_*, *n_cr_*, and *w_cr_*, of HPFRCCs are the functions of *τ_eq_* [51]:

(4) $$\sigma_{pc}=\lambda \cdot \tau_{eq}\cdot \frac{l_{f}}{d_{f}}\cdot V_{f}$$

(5) $$\varepsilon_{pc}≅(n_{cr}\cdot w_{cr})/L$$

(6) $$n_{cr}=L/(\eta \cdot \frac{d_{f}\cdot (1-V_{f})\cdot \sigma_{m}}{\alpha_{2}\cdot V_{f}\cdot \tau_{eq}})$$

(7) $$w_{cr}≅(P_{pc}/A_{f}E_{f})\times (\eta \cdot \frac{d_{f}\cdot (1-V_{f})\cdot \sigma_{m}}{\alpha_{2}\cdot V_{f}\cdot \tau_{eq}})$$

where *λ* is the product of several coefficients related to the type of fiber, *L* is the gauge length of the specimen (=100 mm), *η* is a factor between 1 and 2 for the crack spacing, *σ_m_* is the tensile strength of the matrix, *α*_2_ is a factor describing the fiber distribution, *P_pc_* is the applied force at the post-cracking point, and *E_f_* is the elastic modulus of a fiber.

Thus, single-fiber pullout tests were performed to investigate the reason for the higher *n_cr_* of HPFRCCs containing MGFs (M0.00 and M0.10) compared with the M0.00 series. The equivalent bond strength (*τ_eq_*) can be calculated using Equation (8).

(8) $$\tau_{eq}=\frac{8\times PW}{\pi \times d_{f}\times L_{f}{}^{2}}$$

Here, *d_f_* is the fiber diameter, *L_f_* is the fiber length, and *PW* is the pullout energy, i.e., the area under the curve describing the pullout load versus the slip.

### 4.1. Slump Flow for Uniform Distribution of Both MGFs and Steel Fibers

**Figure 6:**
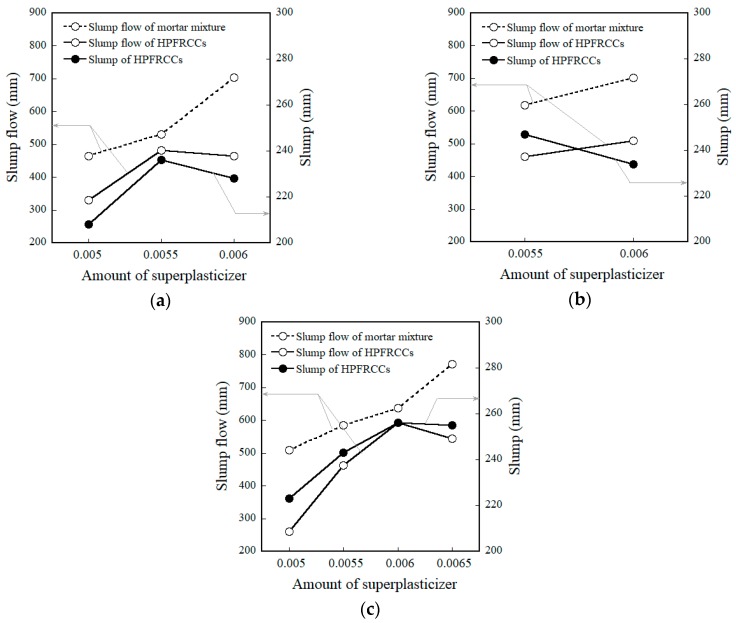
Effects of adding MGFs on the flowability of mortar and HPFRCCs: (**a**) M0.00, (**b**) M0.05, and (**c**) M0.10.

Figure 6 shows the slump flow and slump of the mortar and HPFRCCs, as the amount of superplasticizer increased from 0.005 to 0.0065, corresponding to the amount of MGFs. Figure 6a–c show those for mortar and HPFRCCs containing 0, 5, and 10 wt % MGFs. In Figure 6, the solid lines represent the slump flow and slump of HPFRCCs containing steel fibers, whereas the dotted lines represent the slump flow of the mortar mixture without steel fibers. As the amount of superplasticizer ranged from 0.005 to 0.0065, the slump flow of the mortar mixtures containing only MGFs (except steel fibers) linearly increased regardless of the amount of MGFs. The slump flows of mortar mixtures (M0.00 and M0.05) were measured as 704 and 701 mm, respectively, with a super-plasticizer content of 0.006, reaching a maximum of 772 mm in sample M0.10 containing a superplasticizer content of 0.0065. However, the slump flow and slump of the mortar mixtures containing both MGFs and steel fibers (i.e., HPFRCCs) showed a different tendency. The slump flow and slump of HPFRCCs for M0.00 and M0.10 showed a threshold response to amounts of superplasticizer of 0.0055 and 0.006, respectively. At amounts of superplasticizer greater than these thresholds, the slump flow then decreased for the M0.00 (from 482 to 465 mm) and M0.10 (from 593 to 544 mm) series. The slump also decreased after the superplasticizer thresholds were exceeded for the M0.00 and M0.10 samples. On the other hand, the optimal amount of superplasticizer in M0.05 was determined to be 0.055 because a greater amount (0.006) of superplasticizer resulted in significant fiber accumulation. Thus, the amounts of superplasticizer for a uniform distribution of both MGFs and steel fibers in the mortar matrix were determined to be 0.0055, 0.0055, and 0.006 for the M0.00, M0.05, and M0.10 series, respectively, by considering both the slump flow and slump test results.

### 4.2. Influence of MGFs on the Electromechanical Response of HPFRCCs under Tension

**Figure 7:**
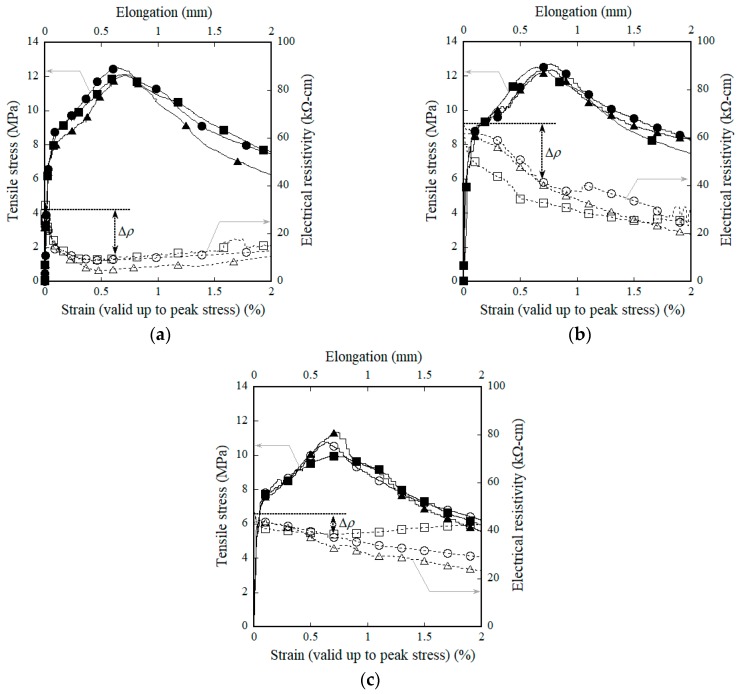
Electromechanical responses of HPFRCCs containing MGFs under tension: (**a**) M0.00, (**b**) M0.05, and (**c**) M0.10.

Figure 7 shows the electromechanical responses of HPFRCCs according to amount of MGFs under direct tension corresponding to differing amounts of MGFs. Figure 7a–c show the results for the M0.00, M0.05, and M0.10 series, respectively. Table 4 and Table 5 summarize the tensile and electromechanical parameters of HPFRCCs containing MGFs. The tensile parameters include the first-cracking strain (*ε_cc_*), the first-cracking strength (*σ_cc_*), the post-cracking strain (*ε_pc_*), the post-cracking strength (*σ_pc_*), and the equivalent number of micro-cracks (*n_cr_*) within the gauge length. The electromechanical parameters include the electrical resistivity at the initial (*ρ*_0_), first-cracking (*ρ_cc_*), and post-cracking points (*ρ_pc_*). The reduction (Δ*ρ*_1_) in the electrical resistivity between the starting point and the first-cracking point and that (Δ*ρ*_2_) between the first- and post-cracking points are also summarized in Table 5. The total reduction in the electrical resistivity (Δ*ρ*) and the normalized reduction in the electrical resistivity per crack (Δ*ρ/n_cr_*) are also summarized in Table 5.

The addition of MGFs to HPFRCCs produced slightly higher *σ_pc_* and *ε_pc_* than that for HPFRCCs without MGFs (as seen in Figure 7), until the amount of MGFs reached 5 wt %. At the addition of 5 wt % MGFs, *σ_pc_* increased from 12.2 to 12.5 MPa while *ε_pc_* increased from 0.68 to 0.72%. However, the addition of 10 wt % MGFs to HPFRCCs clearly reduced *σ_pc_* (10.7 MPa) and *ε_pc_* (0.69%). Moreover, the HPFRCCs containing 5 wt % MGFs also clearly produced a higher *n_cr_* and a higher *ρ*_0_:*n_cr_* was 17.7, 26.2, and 18.3, while *ρ*_0_ was 25.41, 63.11, and 47.15 kΩ·cm for the M0.00, M0.05, and M0.10 series, respectively.

**Figure 8:**
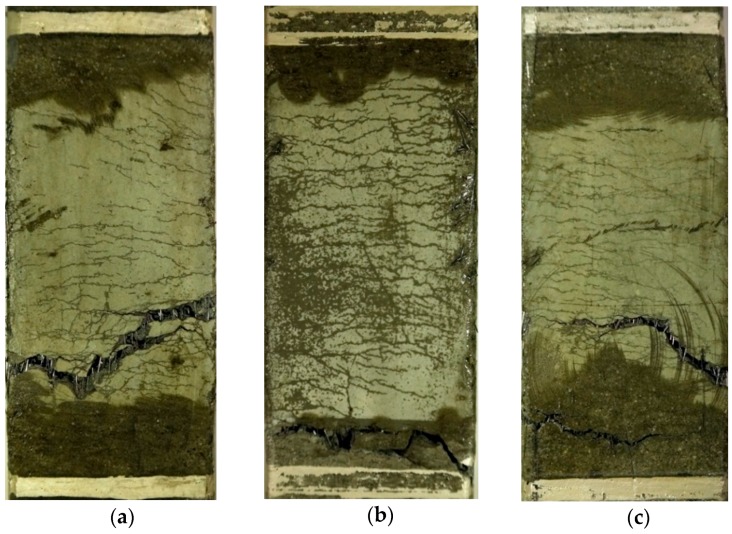
Effects of adding MGFs on the multiple cracking response of HPFRCCs: (**a**) M0.00, (**b**) M0.05, and (**c**) M0.10.

The addition of MGFs produced a notably larger reduction in the electrical resistivity of the HPFRCCs; ∆*ρ* was measured as 17.27, 25.56, and 10.62 kΩ·cm for the M0.00, M0.05, and M0.10 series, respectively. Moreover, the addition of MGFs also produced a distinctly greater reduction in the electrical resistivity in both the elastic and plastic ranges (∆*ρ*_1_ and ∆*ρ*_2_); ∆*ρ*_1_ was 2.10, 2.81, and 3.12 kΩ·cm, while ∆*ρ*_2_ was 15.17, 22.75, and 7.51 kΩ·cm for the M0.00, M0.05, and M0.10 series, respectively. The larger ∆*ρ*_2_ results from the larger *n_cr_* generated during the strain-hardening response, as shown in Figure 8. The addition of MGFs also produced a larger *n_cr_*; the average *n_cr_* of HPFRCCs was calculated as 17.7, 26.2, and 18.3 for the M0.00, M0.05, and M0.10 series, respectively. Moreover, ∆*ρ*/*n_cr_* was 1.12 kΩ·cm for the M0.05 series, which was greater than the reductions observed for the M0.00 and M0.10 series (0.94 and 0.59 kΩ·cm, respectively). Thus, the addition of 5 wt % MGFs to the mixture notably improved the self-damage sensing capacity of HPFRCCs by increasing ∆*ρ*/*n_cr_*.

### 4.3. Influence of MGFs on the Pullout Resistance of Steel Fibers Embedded in Mortar

**Figure 9:**
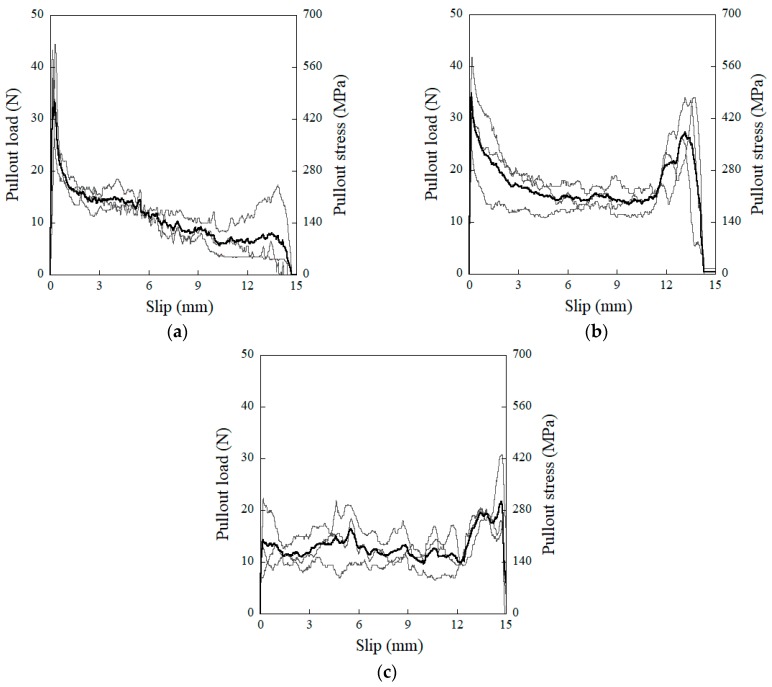
Pullout load (stress) versus slip response of steel fibers embedded in a mortar mixture containing MGFs: (**a**) M0.00, (**b**) M0.05, and (**c**) M0.10.

Figure 9 shows the pullout load (stress) versus slip curves of steel fibers embedded in mortar mixtures containing different amounts of MGFs. The addition of MGFs to the mortar had notable effects on the single-fiber pullout behavior. To quantify how the addition of MGFs influenced the interfacial bond characteristics of steel fibers embedded in mortar, the equivalent bond strengths (*τ_eq_*) were evaluated and summarized (see Table 6).

The *τ_eq_* of the M0.00, M0.05, and M0.10 series was 1.66, 2.12, and 1.98 MPa, respectively. The M0.05 series produced the largest *τ_eq_*, which was strongly correlated with the average crack spacing [52]. The higher *τ_eq_* of steel fibers embedded in the mortar mixture containing MGFs eventually enhanced the self-damage sensing capacity of HPFRCCs by increasing the number of multiple micro-cracks.

## 5. Discussion

**Figure 10:**
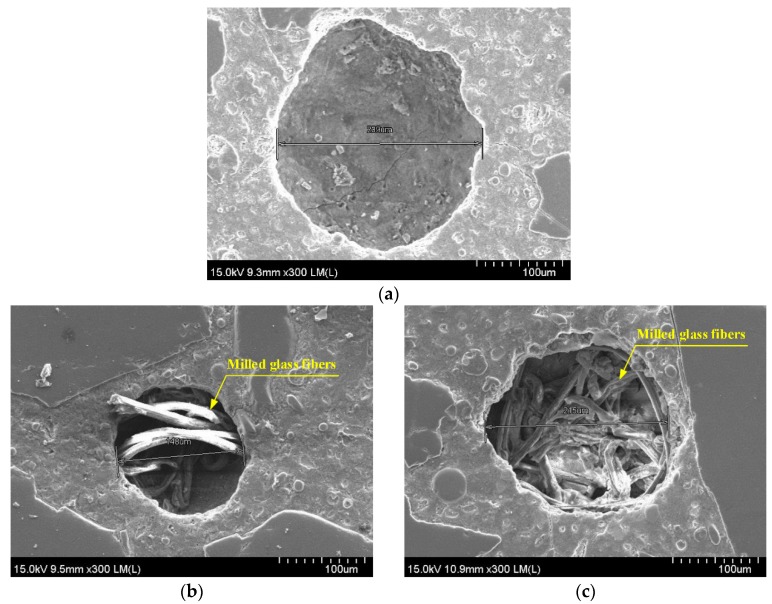
Pores in a mortar mixture containing MGFs: (**a**) M0.00, (**b**) M0.05, and (**c**) M0.10.

**Figure 11:**
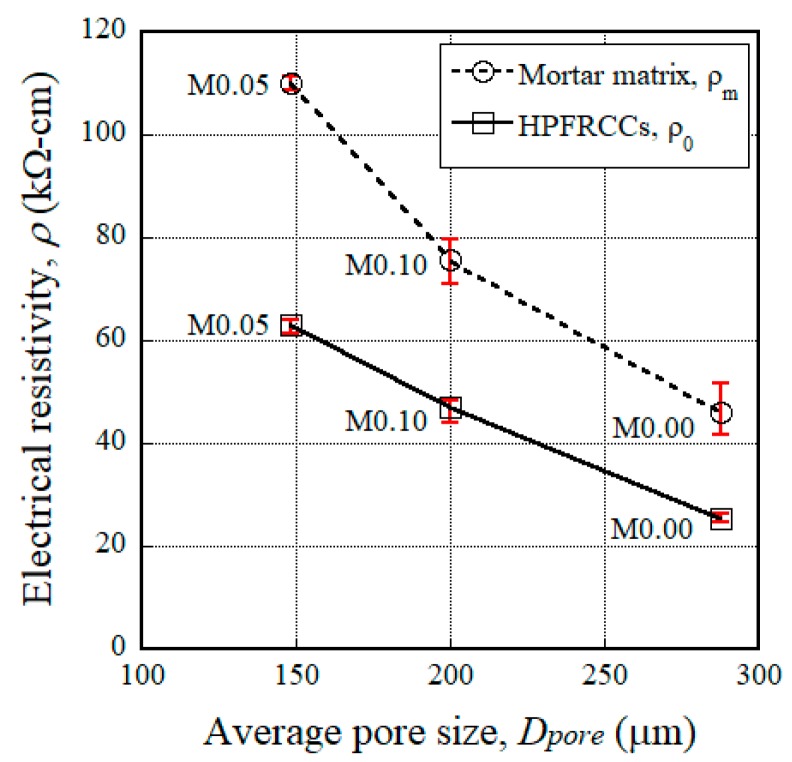
Correlation between the pore size and the electrical resistivity of the mortar matrix.

The results of slump flow and slump tests were found to be important, especially for evaluating the uniform distribution of electrical fillers within the mortar mixture, as this gives materials significantly different electrical resistivities. The amount of superplasticizer required to ensure a uniform distribution of steel fibers in the mixture should be modified according to the amount of MGFs. HPFRCCs showed clearly different electromechanical responses according to the amount of MGFs. Among the mortars and HPFRCCs containing MGFs, the electrical resistivity (*ρ_m_* and *ρ*_0_) was the highest for the M0.05 series (containing 5 wt %), as seen in Figure 2. The higher electrical resistivity of the M0.05 series compared to those of the other series (M0.00 and M0.10), despite containing only half of the content of electrically nonconductive MGFs of the M0.10 series, requires further clarification. Monfore [53] and Teomete [54] reported that the electrical resistivity of a cement-based matrix decreased as the pore size increased since the electrical resistivity of the matrix was greatly influenced by the effective ions within the pores. Thus, by using FE-SEM in this study, we also investigated the pore size in relation to the amount of MGFs (see Figure 10 and Table 7). The average pore sizes were measured as 288.0, 148.3, and 200.3 μm for the M0.00, M0.05, and M0.10 series, respectively. The M0.05 series—with the highest electrical resistivity (*ρ_m_* and *ρ*_0_)—was found to have the smallest pore size (see Figure 11). It has been reported that the pore size influences the electrical properties of cement-based materials [55], even though the distribution of electrically conductive or nonconductive fillers in the mixture is critical for their electrical and/or mechanical properties [15,22,27,30].

**Figure 12:**
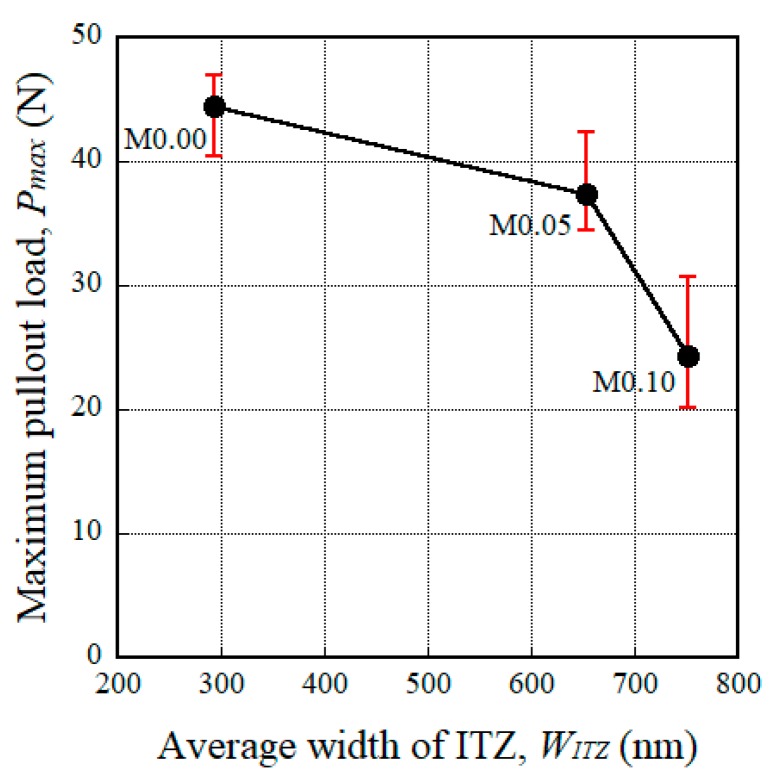
Effect of the width of the ITZ on the maximum pullout load.

**Figure 13:**
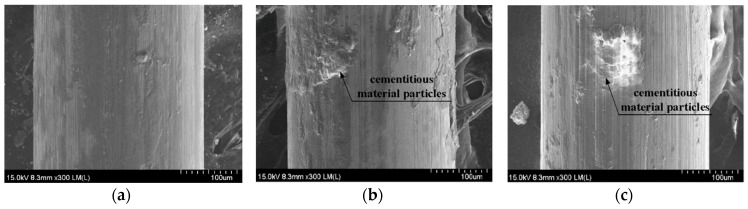
Field-emission scanning electron microscopy (FE-SEM) images of steel fiber surfaces after pullout: (**a**) M0.00, (**b**) M0.05, and (**c**) M0.10.

It is well-known that the interfacial bond resistance between a steel fiber and the mortar is strongly dependent upon the properties of the interfacial transition zone (ITZ), including its width, local stiffness, and porosity. Bentur et al. [56] also reported that the ITZ between the fiber and mortar matrix has a significant effect on the interfacial bond strength. Thus, we also measured the width of the ITZ (*W_ITZ_*) and correlated this with the maximum pullout load (*P_max_*) corresponding to the amount of MGFs (see Figure 12). As the amount of MGFs increased from 0 to 10 wt %, *P_max_* decreased from 41.93 to 24.35 N, whereas *W_ITZ_* increased from 294 to 752 nm. Kong et al. [57] reported that an increased glass fiber content decreased the calcium-silicate-hydrate (C-S-H) gel, leading to a reduced *W_ITZ_*. Moreover, there is a noticeable difference—especially at the end of the fiber pullout curves—according to the addition of MGFs, as can be seen in Figure 9. The pullout load of the M0.00 series showed a continuous decrease after the peak point and then slightly increased at the end of the curve. However, for both the M0.05 and M0.10 series, the pullout load significantly increased again at the end of the curve. As seen in Figure 13, both of the M0.05 and M0.10 series contained many more cementitious material particles attached to the surfaces of steel fibers after fiber pullout than did the M0.00 series. Moreover, the M0.05 series also showed many more such cementitious material particles than the M0.10 series. During fiber pullout, interfacial failure occurred at the interface between the fiber and the matrix in the M0.00 series; however, in both the M0.05 and M0.10 series, failure would have occurred at the matrix rather than at the interface. The cementitious material particles attached to the surfaces of steel fibers accumulated at the interfacial tunnel during fiber pullout; consequently, the pullout load significantly increased at the end of the curves. Thus, the M0.05 series produced the highest *τ_eq_*, which is correlated with the direct tensile response of HPFRCCs. Furthermore, as can be seen in Figure 1, unlike ordinary SFRCs, in which the electrical resistivity showed a slight increase after post-cracking due to the absence of further matrix cracking, the electrical resistivity of HPFRCCs slightly declined even after post-cracking because the cementitious materials were gradually removed from the surface of the steel fibers owing to failure at the matrix rather than at the interface.

**Figure 14:**
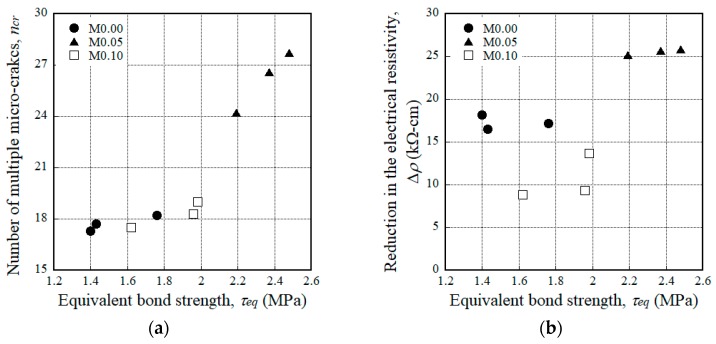
Effects of the equivalent bond strength on (**a**) the number of multiple micro-cracks and (**b**) the reduction in the electrical resistivity.

The mechanical parameters (*σ_pc_*, *ε_pc_*, *n_cr_*) and width of the cracks (*w_cr_*) of HPFRCCs are the functions of *τ_eq_*, as described in Equations (4)–(7). Moreover, the reduction in the electrical resistance of HPFRCCs during the tensile strain-hardening response is affected by *n_cr_*, as described in Equation (2). As *τ_eq_* increased, both *σ_pc_* and *n_cr_* of the HPFRCCs increased. Since the M0.05 series produced the largest *τ_eq_* (2.12 MPa) in the single-fiber pullout tests, the addition of 5 wt % MGFs to HPFRCCs consequently generated the highest *n_cr_* (26.2) under tension, as seen in Figure 14a. Consequently, the M0.05 series produced the largest reduction in the electrical resistivity (Δ*ρ*), as seen in Figure 14b.

## 6. Conclusions

The electromechanical response of HPFRCCs containing MGFs was investigated for the development of a smart construction material with a high self-sensing capacity and low cost. The addition of 5 wt % MGFs to HPFRCCs notably increased the mechanical resistance and self-sensing capacity of HPFRCCs. The following conclusions are drawn from the experimental results:

- The addition of MGFs to cement-based composites reduced the size of pores, which is closely related to the electrical resistivity of the composites; the electrical resistivity was the greatest for HPFRCCs containing 5 wt % MGFs.
- The maximum pullout load (*P_max_*) of steel fibers with a 15-mm embedment length decreased from 41.93 to 24.35 N as the amount of MGFs increased from 0 to 10 wt %, because the width of the ITZ (*W_ITZ_*) increased from 294 to 752 μm.
- However, the equivalent bond strength (*τ_eq_*) of steel fibers, which is closely correlated with the tensile response (especially the number of multiple cracks) of HPFRCCs, increased from 1.53 to 2.35 MPa as the amount of MGFs increased from 0 to 5 wt % owing to the greater accumulation of cementitious material particles attached to the surfaces of steel fibers at the interfacial tunnel.
- The reduction in the electrical resistivity (∆*ρ*) of HPFRCCs during the strain-hardening response under tension was the greatest with the addition of 5 wt % MGFs to the matrix; this was attributed to the increased electrical resistivity of the mortar matrix due to the generation of a greater number of multiple micro-cracks.
- HPFRCCs containing 5 wt % MGFs generated the largest reduction in the electrical resistivity per crack (∆*ρ*/*n_cr_*) of 1.12 kΩ·cm, i.e., the greatest self-damage sensing capacity.

## Figures and Tables

**Table 1:** Matrix composition by weight ratio and properties.

Series	Cement (Type III)	Milled Glass Fiber	Silica Sand	Fly Ash	Superplasticizer ^†^	Water	*f’_ck_* (MPa)	*ρ_m_* (kΩ·cm)
M0.00	1.0	0.00	1.0	0.15	0.005–0.006 (0.0055)	0.35	95	45.9
M0.05	0.95	0.05			0.0055–0.006 (0.0055)		89	110.3
M0.10	0.90	0.10			0.005–0.0065 (0.006)		83	75.8

^†^ The solid content of superplasticizer is 25%.

**Table 2:** Properties of fibers.

Fiber Type	Length, *l_f_* (mm)	Diameter, *d_f_* (mm)	Density (g/cm^3^)	Tensile Strength (MPa)	Elastic Modulus (GPa)	Aspect Ratio, *l_f_*/*d_f_*
Long smooth steel fiber	30	0.3	7.90	2447	200	100
Medium smooth steel fiber	19.5	0.2	7.90	2942	200	97.5
Milled glass fiber	0.3	0.0135	0.58	3367	0.76	22.2

**Table 3:** Chemical components of milled glass fibers (MGFs).

Chemical Composition	Content (wt %)
Silicon dioxide (SiO_2_)	54
Calcium oxide (CaO)	17
Aluminum oxide (Al_2_O_3_)	13
Boron oxide (B_2_O_3_)	6
Magnesium oxide (MgO)	6
Sodium oxide (Na_2_O)	1
Fluorine (F_2_)	0.75
Fluorine oxide (F_2_O_3_)	0.75
Titanium oxide (TiO_2_)	0.75
Strontium oxide (SrO)	0.75

**Table 4:** Tensile parameters of HPFRCCs containing MGFs.

Notation	No.	Tensile Strain (%)		Tensile Stress (MPa)		Equivalent Number of Cracks, *n_cr_*
		*ε_cc_*	*ε_pc_*	*σ_cc_*	*σ_pc_*	
M0.00	SP1	0.024	0.64	5.6	12.5	17.7
	SP2	0.025	0.70	5.6	12.1	17.3
	SP3	0.025	0.70	5.5	12.1	18.2
	***Aver.*** ^a^	***0.025***	***0.68***	***5.6***	***12.2***	***17.7***
	STD ^b^	0.00	0.03	0.05	0.19	0.37
M0.05	SP1	0.030	0.76	6.1	12.7	27.7
	SP2	0.027	0.64	5.8	12.5	24.2
	SP3	0.028	0.77	6.2	12.4	26.6
	***Aver.***	***0.028***	***0.72***	***6.0***	***12.5***	***26.2***
	STD	0.00	0.06	0.17	0.12	1.46
M0.10	SP1	0.020	0.64	5.7	10.8	18.3
	SP2	0.019	0.70	4.9	10.0	17.5
	SP3	0.029	0.73	5.4	11.4	19.0
	***Aver.***	***0.023***	***0.69***	***5.3***	***10.7***	***18.3***
	STD	0.00	0.04	0.33	0.57	0.61

^a^ Aver.: average value; ^b^ STD: standard deviation.

**Table 5:** Electromechanical parameters of HPFRCCs containing MGFs.

Notation	No.	Electrical Resistivity (kΩ·cm)			Reduction in the Electrical Resistivity (kΩ·cm)			Δ*ρ/n_cr_*(kΩ·cm)
		*ρ*_0_	*ρ_cc_*	*ρ_pc_*	Δ*ρ*	Δ*ρ*_1_	Δ*ρ*_2_	
M0.00	SP1	26.25	24.13	9.12	17.13	2.12	15.01	0.94
	SP2	26.30	23.72	9.83	16.47	2.59	13.89	0.93
	SP3	23.67	22.08	5.47	18.20	1.58	16.61	0.95
	***Aver.***	***25.41***	***23.31***	***8.14***	***17.27***	***2.10***	***15.17***	***0.94***
	STD	1.23	0.89	1.91	0.71	0.41	1.12	0.01
M0.05	SP1	65.04	63.45	39.86	25.18	1.59	23.60	0.91
	SP2	60.06	55.65	34.39	25.67	4.41	21.26	1.06
	SP3	64.23	61.80	38.41	25.83	2.43	23.40	1.39
	***Aver.***	***63.11***	***60.30***	***37.55***	***25.56***	***2.81***	***22.75***	***1.12***
	STD	2.18	3.36	2.31	0.28	1.18	1.06	0.20
M0.10	SP1	47.01	44.70	38.22	8.80	2.31	6.49	0.50
	SP2	48.02	43.73	38.66	9.36	4.29	5.07	0.54
	SP3	46.40	43.66	32.69	13.71	2.75	10.96	0.72
	***Aver.***	***47.14***	***44.03***	***36.52***	***10.62***	***3.12***	***7.51***	***0.59***
	STD	0.67	0.47	2.72	2.19	0.85	2.51	0.10

**Table 6:** Pullout parameters of steel fibers embedded in a mortar mixture containing MGFs.

Notation	No.	Maximum Pullout Load, *P_max_* (N)	Equivalent Bond Strength, *τ_eq_* (MPa)	Pullout Energy, *PW* (N·mm)
M0.00	SP1	44.50	1.40	148.54
	SP2	37.95	1.76	186.25
	SP3	43.35	1.43	151.93
	***Aver.***	***41.93***	***1.53***	***162.24***
	STD	2.86	0.16	17.03
M0.05	SP1	41.85	2.48	262.82
	SP2	34.55	2.37	251.59
	SP3	34.00	2.19	232.07
	***Aver.***	***36.80***	***2.35***	***248.83***
	STD	3.58	0.12	12.70
M0.10	SP1	30.80	1.96	207.93
	SP2	20.25	1.62	171.45
	SP3	22.00	1.98	209.42
	***Aver.***	***24.35***	***1.85***	***196.27***
	STD	4.62	0.17	17.56

**Table 7:** Pore sizes and widths of the interfacial transition zone (ITZ).

Notation	Position No.	Pore Size, *D_pore_* (μm)	Width of the ITZ, *W_ITZ_* (nm)
M0.00	Pos.1	395	239
	Pos.2	247	222
	Pos.3	336	337
	Pos.4	174	376
	***Aver.***	***288***	***294***
	STD	84	65
M0.05	Pos.1	138	379
	Pos.2	148	912
	Pos.3	169	595
	Pos.4	138	731
	***Aver.***	***148***	***654***
	STD	13	195
M0.10	Pos.1	215	397
	Pos.2	204	171
	Pos.3	195	1180
	Pos.4	187	1260
	***Aver.***	***200***	***752***
	STD	10	476
